# Genome-Wide Association Mapping of Virulence Genes in Wheat Karnal Bunt Fungus *Tilletia indica* Using Double Digest Restriction-Site Associated DNA-Genotyping by Sequencing Approach

**DOI:** 10.3389/fmicb.2022.852727

**Published:** 2022-05-13

**Authors:** Mohamad Ayham Shakouka, Malkhan Singh Gurjar, Rashmi Aggarwal, Mahender Singh Saharan, Robin Gogoi, Naresh Bainsla Kumar, Shweta Agarwal, Tej Pratap Jitendra Kumar, Bassam Bayaa, Fateh Khatib

**Affiliations:** ^1^Division of Plant Pathology, Indian Agricultural Research Institute ICAR-IARI, New Delhi, India; ^2^Division of Plant Protection, University of Aleppo, Aleppo, Syria; ^3^Division of Genetics, Indian Agricultural Research Institute ICAR-IARI, New Delhi, India

**Keywords:** ddRAD-GBS genotyping by sequencing, GWAS-genome wide association study, SNP, association mapping, virulence genes, *T. indica*, population structure

## Abstract

*Tilletia indica* is a quarantine fungal pathogen that poses a serious biosecurity threat to wheat-exporting countries. Acquiring genetic data for the pathogenicity characters of *T. indica* is still a challenge for wheat breeders and geneticists. In the current study, double digest restriction-site associated-DNA genotyping by sequencing was carried out for 39 *T. indica* isolates collected from different locations in India. The generated libraries upon sequencing were with 3,346,759 raw reads on average, and 151 x 2 nucleotides read length. The obtained bases per read ranged from 87 Mb in Ti 25 to 1,708 Mb in Ti 39, with 505 Mb on average per read. Trait association mapping was performed using 41,473 SNPs, infection phenotyping data, population structure, and Kinship matrix, to find single nucleotide polymorphisms (SNPs) linked to virulence genes. Population structure analysis divided the *T. indica* population in India into three subpopulations with genetic mixing in each subpopulation. However, the division was not in accordance with the degree of virulence. Trait association mapping revealed the presence of 13 SNPs associated with virulence. Using sequences analysis tools, one gene (*g4132*) near a significant SNP was predicted to be an effector, and its relative expression was assessed and found upregulated upon infection.

## 1. Introduction

The virulence of fungal plant pathogens is complex and difficult to be characterized at the genetic level, due to several factors including the high level of genetic diversity among genotypes in the natural populations, the complexity of inheritance between pathogens' generations, the difficulties in conducting crosses among different genotypes for association mapping, the scarcity of genomic information, and the high cost of genotyping using *de novo* sequencing (Kumar et al., 2016). However, obtaining high density genotyping methods can circumvent these limitations, especially since, the conventional markers only revealed a weak link between genotypes and virulence traits (Kolmer, 2001, 2013, 2015; Kolmer and Acevedo, 2016). The 6 k Illumina Infinium single nucleotide polymorphism (SNP) assay is considered the first high dense genotyping tool (Song et al., 2015), which has been used in many bi-parental mapping studies and helped in discovering many important agronomic traits. However, fast genotyping of hundreds or thousands of SNPs is not feasible using it (Bello et al., 2014; Brisco et al., 2014; Mukeshimana et al., 2014). Recently, a new high-density genotyping method called genotyping by sequencing (GBS) has become available, in which, next-generation sequencing (NGS) is combined with restriction-associated DNA (RAD) tagging that successfully generated a large number of SNPs per population, which could be used in the mapping of particular genomic regions (Moghaddam et al., 2016), and enabled multiple individuals to be sequenced at the same time (Mascher et al., 2013). This method employs restriction enzymes to capture genomic regions of interest, followed by the adaptors' ligation to the flanking sections of the restriction sites, resulting in barcoded libraries. HiSeq and Illumina GAII platforms were primarily employed in this method (Poland and Rife, 2012). Recently, an enhanced RAD-GBS methodology adopted for the Ion Torrent has just been available (Rothberg et al., 2011). RAD-GBS has been successfully employed in genotyping a variety of plant traits (Leboldus et al., 2015; Gao et al., 2016; Carlsen et al., 2017), generating high-density genotypes datasets by utilizing Illumina NGS technologies (Baird et al., 2008; Baxter et al., 2011; Chutimanitsakun et al., 2011; Elshire et al., 2011; Pfender et al., 2011; Ma et al., 2012; Poland et al., 2012a). Targeting certain genomic regions can result in a significant number of (SNP) markers that can be utilized to create a lower-complexity genome sequence (Elshire et al., 2011; Poland et al., 2012b), and gives a plethora of genetic data that may be used to determine the relationship between desired phenotypes and genetic markers using recombination events based on linkage disequilibrium (LD) accumulated over many generations (Korinsak et al., 2019). Furthermore, this approach has been employed in a variety of investigations, including genetic mapping, genomic selection, elucidating the basis of disease resistance related traits at the genetic level, and genetic variability studies in a variety of taxa (Adhikari and Missaoui, 2019). Using the generated genotyping data in genome-wide association studies (GWASs) identified thousands of genetic loci associated with complex phenotypes and had advantages over biparental mapping in terms of map resolution and time to build a mapping population (Welter et al., 2014). Plants have long employed association mapping using this approach, and fungi have lately been added to the list. In *Puccinia triticina*, 30 isolates collected from different locations were genotyped based on 2,125 SNP markers discovered using the ddRAD approach, phylogenetical analysis grouped the studied isolates in nine distinct clusters (Aoun et al., 2020) with a general correlation between the genotyping data and virulence phenotyping data. Similarly, genome wide association study of 73 isolates of *Magnaporthe oryzae* demonstrated the presence of eight subpopulations, however, their division was unrelated to the degree of virulence. Association mapping analysis revealed that five SNPs were found associated with fungal virulence in the studied population (Korinsak et al., 2019). The availability of such a method can suggest a sufficient number of SNP markers (Poland and Rife, 2012) making the information on pathogenicity profiles of the pathogen more available. A detailed understanding of the genetic makeup and genes governing pathogenicity is essential to provide the required significant data for use in effective management strategies of some pathogens, particularly those for which genomic information is still scarce. *Tilletia indica* is a quarantine fungus causing Karnal bunt of wheat in many tropical countries, a characteristic rotting- fish like odor (due to the presence of trimethylamine) liberates from infected grains. The main economic effect of Karnal bunt disease is a reduction in grain quality rather than quantity. It reduces the viability and weight of the seed, as well as the quality of the flour (Singh, 2005). High virulence and genetic variability were detected among *T. indica* isolates due to the secondary sporidia mating immediately before the infection resulting in genetic recombination (Gurjar et al., 021a; Kumar et al., 2009; Aggarwal et al., 2010). The knowledge about the genomic regions linked with *T. indica* virulence is still limited, and obtaining such information is essential for the identification and characterization of virulence-related traits, as well as their regulatory genes and their corresponding resistance related genes. Determining which virulence genes exist in each population will also aid in the formulation of a breeding program based on the usage of optimal resistance genes. Accordingly, the present study was performed with a view to executing a genome-wide association study in *T. indica* using a ddRADseq-genotyping by sequencing approach to identify SNPs and virulence factors in the population of *T. indica* in India.

## 2. Materials and Methods

### 2.1. Fungal Population

Infected wheat grains were collected from 33 different locations in India (Supplementary Table S1) and bunted kernels were detected by visual examination and then separated. The fungal cultures were established following the Warham method (Warham, 1986). Briefly, 20 μl of the spore suspension from each sample was placed into 2 ml centrifuge tubes, the volume was made up to 2 ml with sterile distilled water and kept overnight inside an incubator at 21°C to moisten the harvested teliospores, and increased the susceptibility of contaminating saprophytes to sterilization. The tubes were then centrifuged at 1,200 g for 3 min, and the supernatants were discarded. For surface sterilization, the pellets were suspended in 5 ml of (0.3–0.5% NaOCl), reversing the tubes several times, and then centrifuged at 1,200 g for 1 min (Bonde et al., 1999). The supernatant was discarded and the pellets were washed two times with 1 ml of sterile distilled water and centrifuged at 1,200 g for 5 min. Finally, 1 ml of sterile distilled water (SDW) was used to resuspend the pellets and 200 μl of the spores suspension was inoculated into 2 % water agar (WA) plates containing streptomycin sulfate (Bulletin, 2007). The water agar plates were kept inside an incubator at 18 ± 2°C with a 12-h light cycle and checked regularly under a microscope until the teliospores germinated and produced promycelium bearing basidiospores. Upon germination, a small bit of media (1 x 1 cm) having a germinated teliospore was transferred and stuck to the underside of a Petri plate cover, which allowed the sporidia to be deposited onto the media surface. The inoculated plates were then kept inside an incubator at 18 ± 2°C with a 12-h light cycle until mycelial mats appeared on the media surface, thereafter it was transferred into a test tube containing PDA media, labeled, and incubated at 18 ± 2°C with a 12 h light cycle, with subculturing on regular time intervals of 3 weeks.

### 2.2. Assessment of Karnal Bunt Disease and Virulence Analysis

Seeds of susceptible wheat genotype (WH542) were sown in 12-inch pots under the net house conditions during the winter season (November–March). To make the inoculum, sterile distilled water was poured over the fungal slants cultures and scraped gently with a glass rod to liberate allantoid secondary sporidia. To remove media debris, the spore suspension was filtered through a double layer of muslin fabric, and the spore concentration was set at more than 10,000 sporidia per ml. Five healthy plants per pot were inoculated at the booting stage (Z-49 stage) (Zadoks et al., 1974) by syringe inoculation method in the evening hours (Aujla et al., 1987) under conditions of excessive humidity. The inoculated heads were plucked and threshed individually as they reached maturity. The endosperm portion that had developed into a sooty mass of teliospores was utilized to classify the infected grains into five infection categories (Table 1).

**Table 1:** grades given to Karnal bunt infected wheat grains.

**Grades**	**Description**
0	Healthy seed of wheat
1	Germinal tip infection
2	Infection advancing to the kernel groove (1/2th grain)
3	More advanced infection (3/4th of the grain)
4	“Canoe” symptom-the hollowing out of seed interior

The coefficient of infection was calculated as per the method of Aujla et al. (1989).


(1) $$\begin{array}{r} coefficient\;of\;infection=\left(XiYi/N\right)100 \end{array}$$

Where, Xi and Yi refer to numerical ratings and grains counts, respectively and i is a value between 0 and 4 (grade of infection).

### 2.3. Genomic DNA Extraction and Double Digest Restriction-Site Associated DNA- Genotyping by Sequencing

Liquid potato dextrose broth media was used to establish the fungal colonies. Mycelial mats were gathered once they appeared using sterile filter papers and preserved at −80°C. The CTAB method was used to extract the DNA (Crowhurst et al., 1995). Extracted DNA samples (50 μl) were then incubated for one hour at 37°C with 2 μl of RNase-A. The concentration and purity of DNA were evaluated using UV absorbance at 260 and 280 nm in a Nano Drop 2000/2000 (Thermo Scientific) as well as electrophoresis on agarose gels with a marker (1 kb DNA ladder, Thermo Fisher Scientific, USA) and visualized with a UV transilluminator. GBS libraries were created following Peterson's approach (Peterson et al., 2012). Briefly, using the restriction enzymes *SphI* and *MluCI* double digestion of genomic DNA (1 μg) of each *T. indica* isolate was conducted. After that, Ampure beads were used to clean the digested products before they were ligated using T4 DNA ligase with the forward and reverse P1 and P2 barcoded adaptors. The ligated products were then pooled, cleaned, and size selected using 2% agarose gel electrophoresis (Thermo Fisher, Waltham, MA, USA). PCR amplification was carried out to enrich and add the Illumina specific adapters and flow cell annealing sequences. The quality was checked on a bioanalyzer high sensitivity DNA kit (Agilent Technologies, Santa Clara, CA, USA), followed by final pooling and sequencing in AgriGenome Labs Pvt Ltd. Upon sequencing, indexes were removed from raw and checked for the presence of rad-tags on both reads using in-house scripts. The dDocent (v.2.6.0) was used to remove any low-quality bases (below a quality score of 20) and adapters (Puritz et al., 2014), a sliding 5 bp window was used to trim the bases when the average quality score drops below 10. After quality filtering, filtered reads took by the dDocent pipeline for alignment using BWA (v-0.7.8). The alignment has been done with filtered reads against the reference genome *T. indica* RAKB–UP–1 ASM222083v1. The SNPs were called with Freebayes (v.1.2.0), and the bi-allelic SNPs were filtered with bcftools (v.1.6) and Agri Genome Inhouse scripts at read depths of 5 and 10. The homozygous polymorphic marker identification was carried out using the bi-allelic genotype at read depth 10. The kinship matrix was generated on TASSEL (v 5.2.31) (Bradbury et al., 2007).

### 2.4. Population Structure and Phylogenetic Analysis

The number of possible population structures of *T. indica* was analyzed using the Bayesian clustering approach in STRUCTURE version 2.3.4 (Greenbaum et al., 2016). The number of feasible clusters (K) was checked from 1 to 10, with each K value receiving 10 iteration runs. The admixture model was used to run 1,00,000 Markov Chain Monte Carlo (MCMC) repetitions after a burn-in time of 1,00,000 steps. The *ad hoc* statistic Delta K was used to calculate the ideal K value in STRUCTURE HARVESTER version 0.6.94 (Earl and vonHoldt, 2011). STRUCTURE PLOT 2.0 (Ramasamy et al., 2014) was used to create a graph. DARwin version 6.013 (Bradbury et al., 2007) was used to implement the neighbor-joining cluster analysis and principal coordinate analysis (PCoA). The principal component analysis (PCA) was done using SNP related package in R (Team et al., 2013) based on the identified SNPs.

### 2.5. Virulence Gene Association Mapping

Genome-wide association study (GWAS) was carried out based on the mixed linear model (MLM) to calculate the relationships between markers and phenotyping data using TASSEL software v 5.2.31 (Bradbury et al., 2007). To avoid misleading connections, MLM uses phenotyping data, population structure (Q), genotyping data, and kinship (K) matrix as covariates during a GWAS. The logarithm of odds was calculated based on the *P*-value estimated using TASSEL software v 5.2.31, and significantly associated SNPs were cut if their LOD score was equal to or greater than 3.0. The proportion of the phenotypic variation explained (PVE) was calculated for each marker using the relevant R2 in TASSEL software v 5.2.31.

### 2.6. Gene Annotation

The nucleotides sequence was retrieved from a region of 250 Kb upstream and downstream of the SNPs using a customized R script (Team et al., 2013), and used for candidate genes search. Functional annotations of each postulated candidate gene were investigated using the Swiss-Prot database (Boutet et al., 2016). The candidate genes were run with SignalP 4.0 (Petersen et al., 2011) to obtain the initial list of potential secreted proteins (SPs). TMHMM 2.0 (Krogh et al., 2001) was used to predict potential transmembrane domains. Only proteins with zero or one transmembrane domain prediction that overlapped the signal peptide prediction were considered for further investigation. Protein sub-cellular localizations were predicted using WoLFPSORT (Horton et al., 2007).

### 2.7. *In planta* Relative Expression Analysis of the Predicted Virulence Gene

Under net house conditions, seeds of resistant (HD30) and susceptible (WH542) wheat genotypes were sowed in 12-inch pots (10–15 seeds per pot). Five healthy plants per pot were inoculated at the booting stage (Z-49 stage) (Zadoks et al., 1974) using the syringe method (Aujla et al., 1989). Inoculated heads were harvested at 0, 1, 3, 5, and 13 days after inoculation. Three biological replicates from each treatment were harvested and stored at −80°C. Trizol method (Chomczynski and Mackey, 1995) was used for RNA extraction from the infected spikes. Total obtained RNA was used for cDNA synthesis using the Verso cDNA synthesis kit (Thermo Fisher Scientific). The GenScript Primer Design software (https://www.genscript.com/tools/real-time-pcr-tagmanprimer-design-tool) was used to design the specific primers for the putative effector gene (Forward: 5' AAGAACGCCGAATGGGATTG 3', Reverse: 5' AGAGAGGGCAGACCTTGAAC 3'). *GAPDH* gene was used as a reference gene (Forward: 5' GGGAGCAGAGATGACAACCT 3', Reverse: 5' GAGTCCACCGGTGTCTTCA 3'). GCC Biotech (India) Pvt. Ltd. synthesized the designed primers. The expression of the potential effector was measured using a real-time PCR technique and the fold changes value was calculated using the 2 (“delta delta CT) method (Rao et al., 2013), and statistically analyzed using an unpaired *t*-test (Schober and Vetter, 2019).

## 3. Results

### 3.1. Virulence Analysis of *T. indica* Isolates

**Figure 1:**
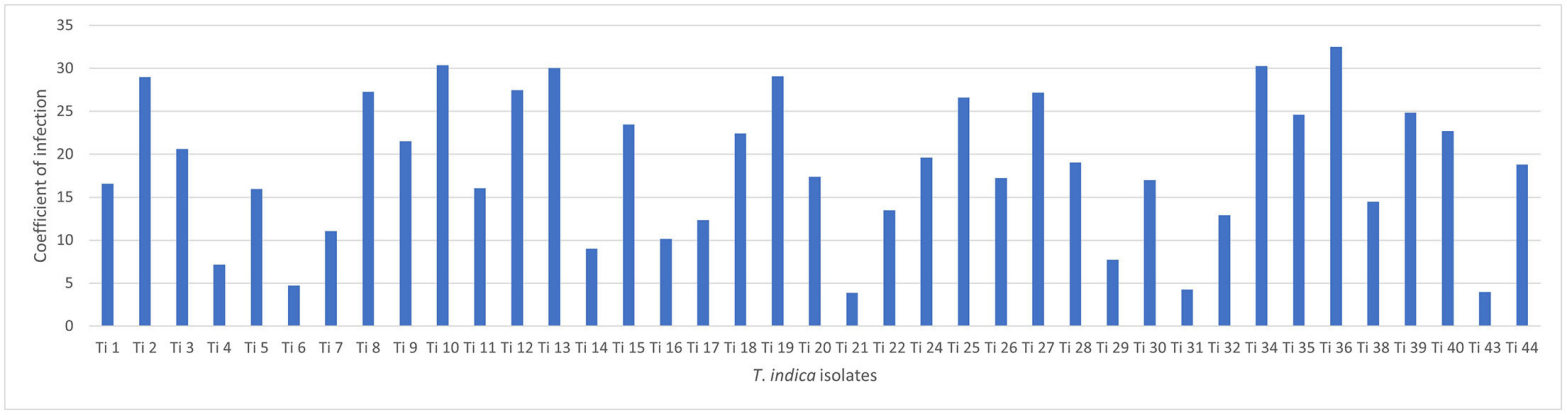
Virulence analysis of *Tilletia indica* isolates.

Successfully, 39 *T. indica* cultures were established and maintained. The virulence analysis on susceptible wheat genotype (WH 542) showed variability in virulence among the isolates. The coefficient of infection (CI) ranged from (3.88) in Ti 21, Tonk, Rajasthan which was less virulent to (32.5) in Ti36, Taroari, Karnal, Haryana which was highly virulent (Figure 1). Among the isolates, 17 isolates showed highly aggressive causes of the disease, most of them were collected from Rajasthan, and one of them, Ti39, was collected during the year 2007 from Meerut, UP, and found still highly virulent (Supplementary Table S1).

### 3.2. Genotyping by Sequencing (dd-RAD-GBS) of *T. indica* Isolates

Double digestion of the extracted genomic DNA from 39 *T. indica* isolates was performed using two restriction enzymes *SphI* and *MluCI*, those generated reduced representation libraries, with 3,346,759 raw reads per sample on average, and 151 x 2 nucleotides read length. The obtained bases per read ranged from 87 Mb in Ti 25 to 1,708 Mb in Ti 39, with 505 Mb on average per read. The sequencing reads were then filtered for the presence of tags on both sides, and reads with tags on both sides ranged from 117,000 reads in Ti 5 to 9,000,000 in Ti 11, with an average of 1,804,287 reads. The data were then cleaned up by removing the low-quality bases and adaptors, resulting in 1,717,146 reads on an average per sample, which has been used for further analysis.

### 3.3. Mapping and Allelic Variation

**Figure 2:**
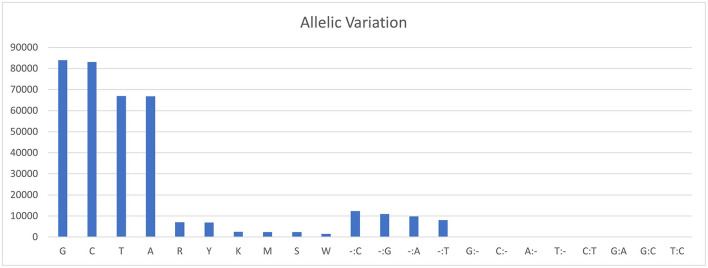
Allelic variations frequency in *T. indica* isolates. Axis-x shows the nucleotide bases and axis-y represents the frequency of each nucleotide.

**Figure 3:**
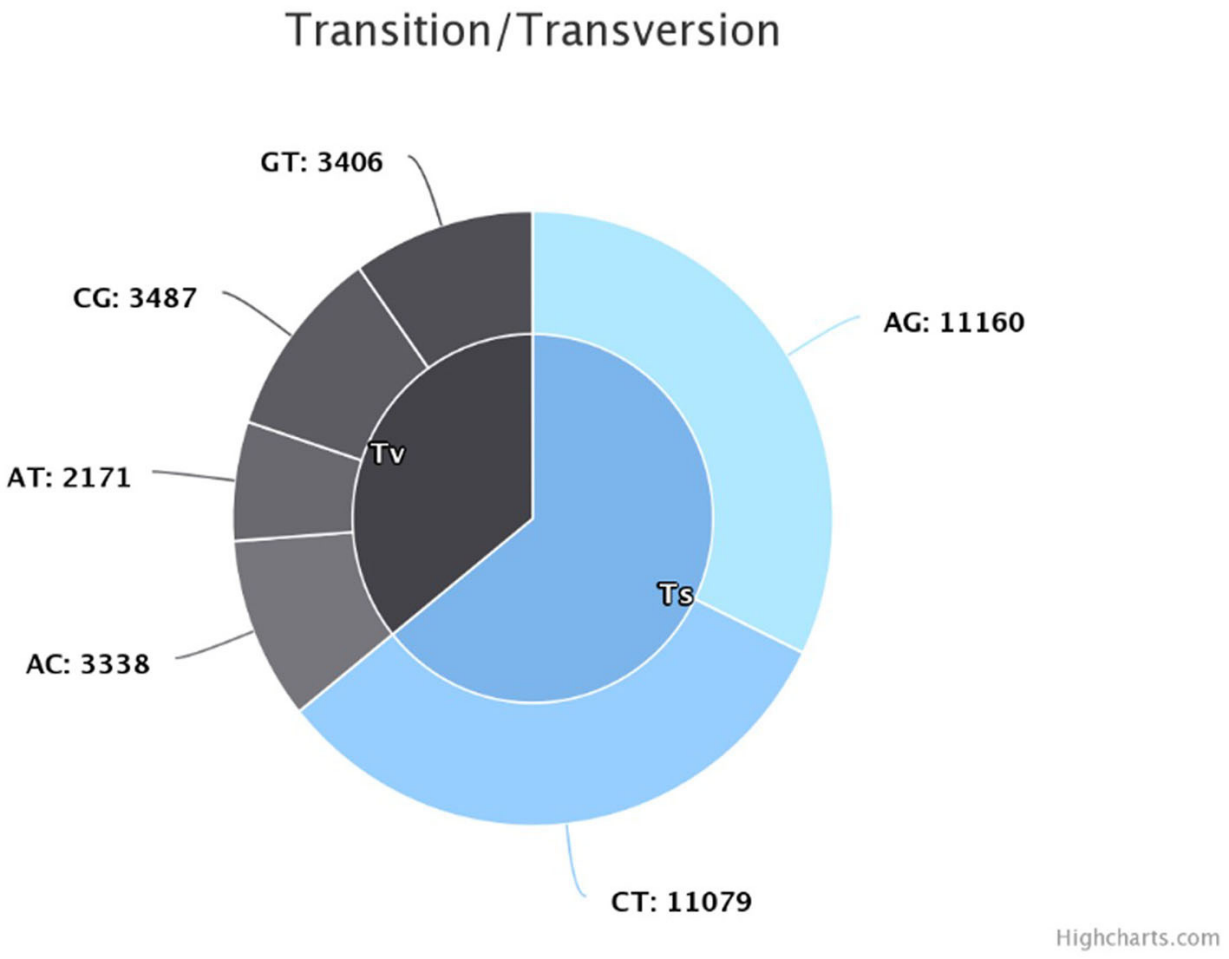
Transition and transversion frequency in the identified single nucleotide polymorphisms (SNPs).

**Figure 4:**
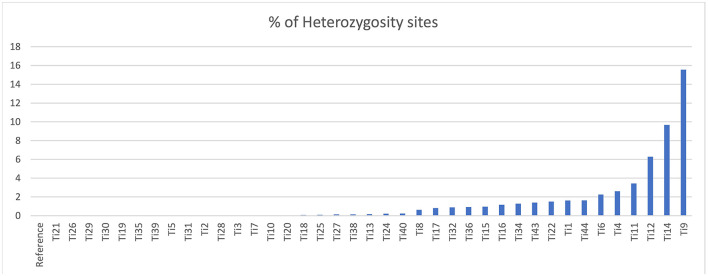
Heterozygosity rates in *T. indica* isolates.

The sequencing reads with quality scores ratings greater than 20% were aligned against the reference genome *T. indica* RAKB–UP–1 ASM222083v1 and revealed the presence of 41,473 SNPs among *T. indica* isolates. The most frequent base in the variation sites was guanine (83,993 times), while the lowest was W (either Adenine or Thiamine) with 1,540 times (Figure 2). The transition and transversion SNPs analysis showed that transition SNPs were more frequent (64.19%, 22,239 SNPs) than transversion ones (35.80%, 12,402 SNPs). The A/G transitions were detected in 11,160 positions, accounting for the highest frequency (32.21%), while A/T transversions occurred in the lowest frequency (6.26%) and were detected in 2,171 positions (Figure 3). The observed heterozygosity ranged from 0.01 to 15.56% with an average of 1.34% (Figure 4).

### 3.4. Minor Allele Frequency Distribution

**Figure 5:**
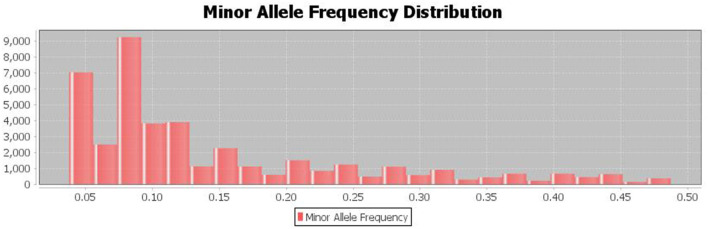
The distribution of minor allele frequencies of the discovered SNPs shows Strong skew toward rare alleles (alleles with frequency <0.1%).

The population's minor allele frequency (MAF) spectrum exhibited a substantial skew toward rare alleles, indicating that there were no recent genetic bottlenecks or mutations and that the detected major alleles for the SNP are preserved (Figure 5).

### 3.5. Population Structure of *T. indica* in India

**Figure 6:**
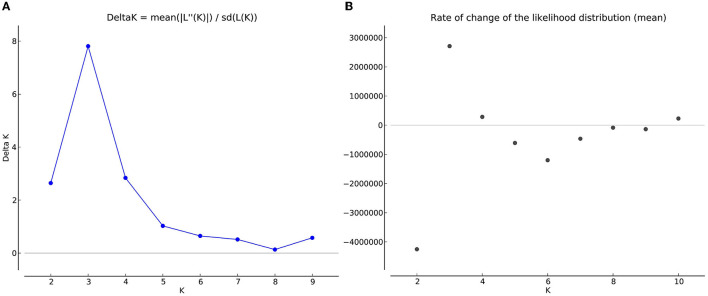
**(A)** Delta K graph obtained by structure Harvester software, a derivative of the mean log-likelihood function of Delta K, showing the highest value of delta K at K=3, which indicated that the entire population of *T. indica* in India can be divided into three sub-population **(B)** The mean log-likelihood of the number of populations (K) run on a set of potential K values (1–10) and assessed through longer Markov chain Monte Carlo (MCMC) generations.

**Figure 7:**
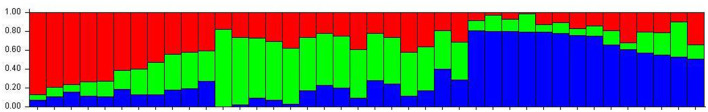
Population structure of 39 isolates of *T. indica* based on 41,473 SNPs using model-based Bayesian. Dividing the population of *T. indica* in India into 3 subpopulations. Individuals are represented as bars, with the color of each bar indicating the degree of genetic mixing between subpopulations.

**Figure 8:**
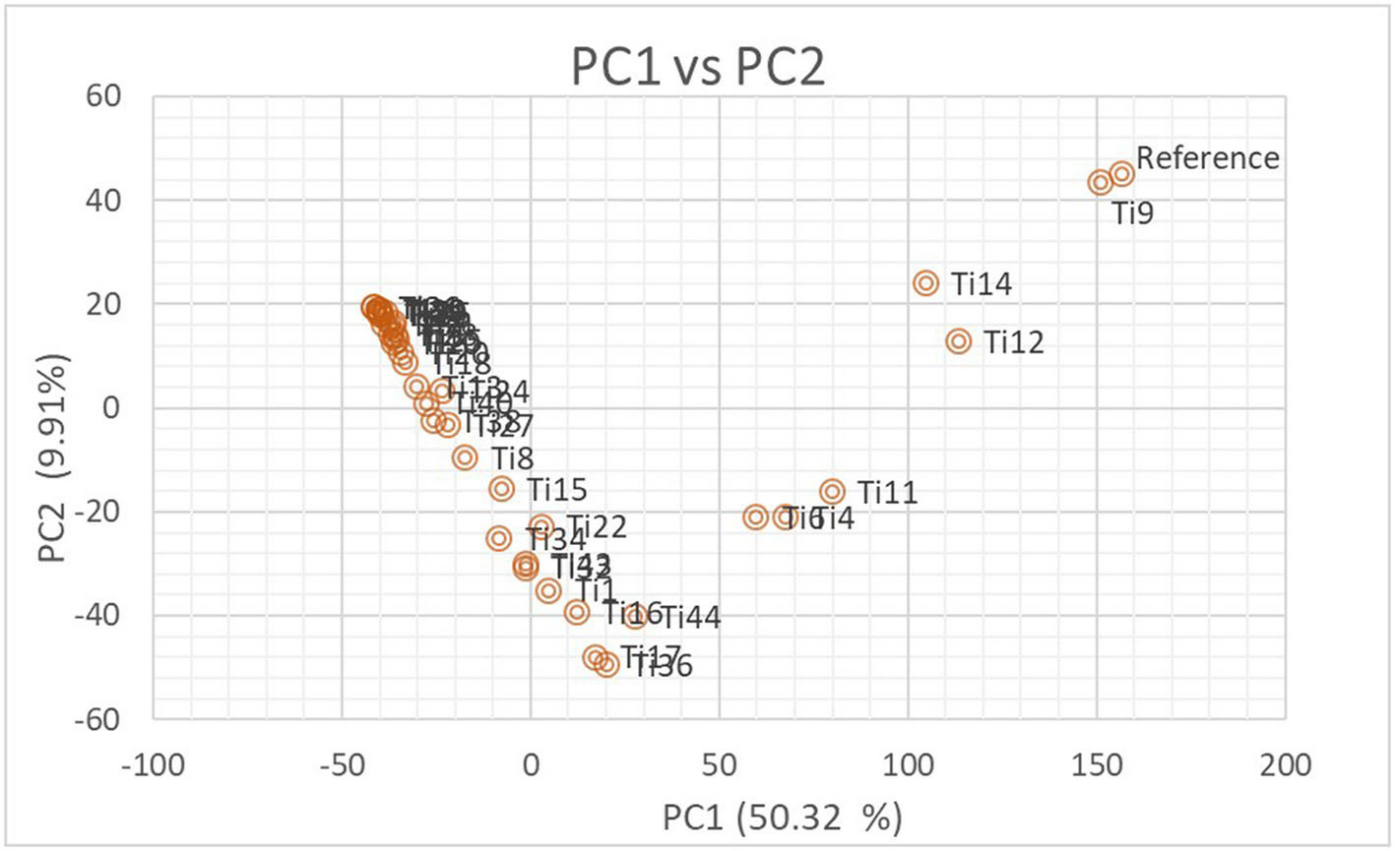
Biplot showing the relationship among *T. indica* isolates in terms of principal components analysis (PCA). Axis-x and axis-y refer to the first and second principal components, which explained 50.32% and 9.91% of the total variation, respectively.

**Figure 9:**
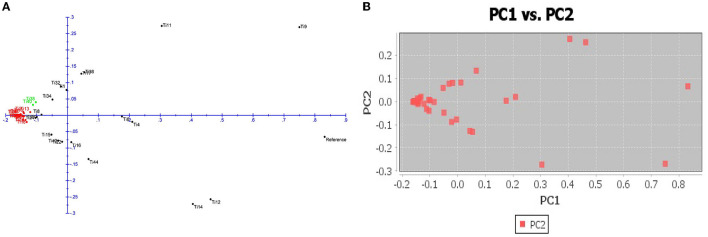
**(A)** Principal coordinate analysis (PcoA) for 39 *T. indica*. The three colors correspond to the three clusters identified by the phylogenetic analysis. **(B)** Multi-Dimensional scaling (MDS) showing variation among the isolates.

The number of subpopulations in the 39 *T. indica* isolates tested was determined using the STRUCTURE 2.3.4 software. The population was analyzed using a total of 41,473 SNPs detected in 39 isolates. To determine the appropriate number of subpopulations, the number of clusters (K) was plotted against ΔK (Figure 6). The highest ΔK value was found at K = 3, indicating that the investigated isolates belong to three subpopulations. As a result, three clusters were the most likely number of clusters present in this population. Clusters ordered by Q (membership coefficient) are shown in Figure 7. Genetic mixing of more than one sub-population with varying proportions was seen in all populations. The three groups consist of the red, blue, and green groups, respectively. PCA showed that the first and the second principal components explained 50.32 and 9.91%, respectively, of the total variability (Figure 8). The first quarter accommodated isolates most of them were collected from Uttar Pradesh, India, and were more virulent as compared to the remaining quadrants. The second quarter accommodated the reference genome along with 3 isolates collected from Rajasthan, India, and all of them were plotted away from the origin. The third and fourth quarters accommodated isolates were collected from Haryana, India, and Rajasthan, India. The Multi-Dimensional scaling (MDS) and PcoA (Figure 9) showed a similar relationship among *T. indica* isolates.

### 3.6. Phylogenetic Tree Analysis in 39 Isolates of *T. indica*

**Figure 10:**
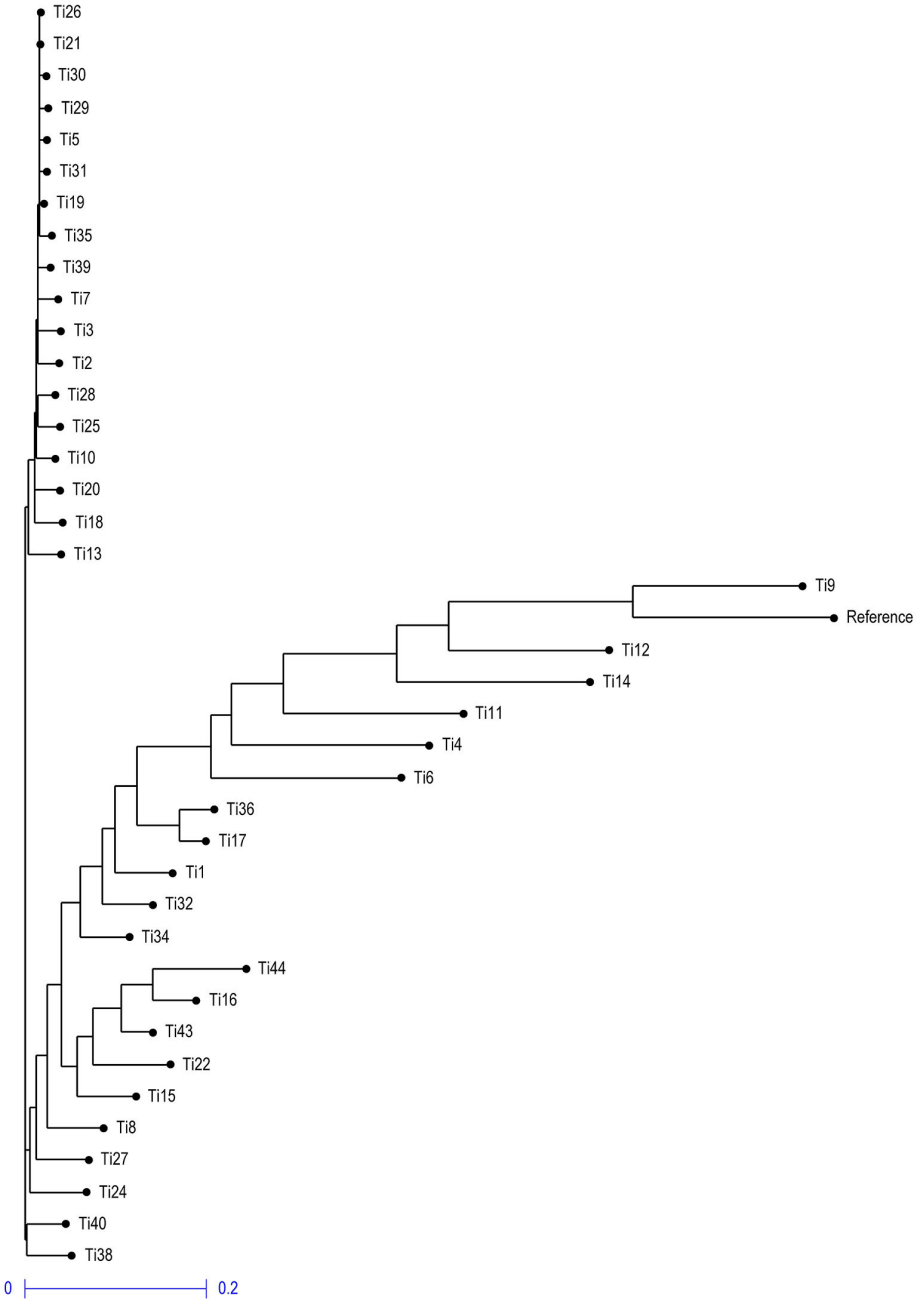
Neighbor-joining phylogenetic tree illustrating the genetic relationship among 39 *T. indica* based on 41,473 SNPs.

Neighbor- joining phylogenetic tree grouped the isolates into three clusters (Figure 10), with a variable number of individuals inside each one. The first cluster included only two isolates Ti38 and Ti40 which were quite distinct from other isolates and belong to Uttar Pradesh, India, while the second cluster included isolates and most of them were collected from Rajasthan, India. The third cluster included all the remaining isolates and most of them were collected from Uttar Pradesh, India.

### 3.7. Trait Association Mapping

**Figure 11:**
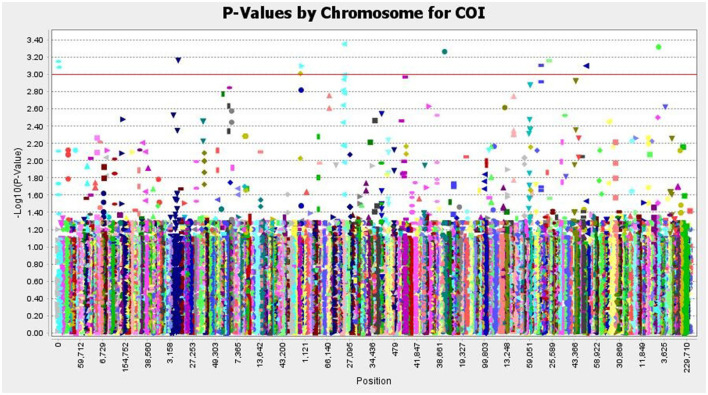
Manhattan plots based on a mixed linear model representing - log^10^ (P-values) for SNPs loci distributed throughout the *T. indica* genome and showing the significant virulence associated markers, The SNP positions and the genome-wide scan - log^10^ (P-values) are shown on the X-axis and the Y-axis, respectively.

The SNP matrix used for association mapping in this study was generated by genotyping by sequencing (GBS) of 39 *T. indica* isolates, followed by GBS “Discovery Pipeline” using TASSEL (v 5.2.31). A total of 41,473 detected SNPs, saved in HapMap were used as genotypic data for GWAS. In order to find virulence genes, the disease reactions on susceptible wheat genotype (WH 542), principal components analysis (PCA), population structure, and Kinship matrix were used to conduct SNP-trait association analysis and revealed significant single nucleotide polymorphisms associated with the disease virulence at 13 positions in the genome (Figure 11 and Table 2).

**Table 2:** The position of significantly associated single nucleotide polymorphisms (SNPs) with virulence of *Tilletia indica* based on association analysis using the mixed linear model (MLM).

Trait (CI^*^)	**Marker**	**Alleles**	**Position**	**MarkerR2**
CI	rs.MBSW01000808.1.55604	C/T	5,5604	0.54587
CI	rs.MBSW01001646.1.3308	G/A	3,308	0.53852
CI	rs.MBSW01001051.1.80074	C/G	80,074	0.52753
CI	rs.MBSW01000359.1.360837	G/A	360,837	0.50702
CI	rs.MBSW01001343.1.21803	T/C	21,803	0.50666
CI	rs.MBSW01001343.1.21832	C/T	21,832	0.50666
CI	rs.MBSW01000006.1.6428	G/A	6,428	0.50505
CI	rs.MBSW01001301.1.21986	C/T	21,986	0.49573
CI	rs.MBSW01001419.1.48293	A/G	48,293	0.49462
CI	rs.MBSW01001419.1.48328	G/A	48,328	0.49462
CI	rs.MBSW01000668.1.18323	C/T	18,323	0.49453
CI	rs.MBSW01000006.1.40825	T/G	40,825	0.49141
CI	rs.MBSW01000660.1.17242	A/C	17,242	0.47701

*CI, Coefficient of infection*.

### 3.8. Identification of Candidate Genes

A search within 500 kb (±250*kb*) around of the peak SNPs identified a total of 43 potential genes near significant SNP (Supplementary Table S3). Gene annotation using Swiss-Prot showed that most of those genes (22 genes) had an uncharacterized function and only 21 genes had functional annotations. The annotated genes had a wide variety of functions, 12 genes were found to have putative virulence functions. To discover potential effectors, the identified genes were analyzed using sequence-based prediction tools. Out of the 43 genes, 12 genes were predicted to secrete proteins have a signal peptide. The potential transmembrane domains were also predicted and only 7 genes were found to have no or only one transmembrane domain protein overlapping the signal peptide prediction, and these genes were further investigated. Subcellular localization using revealed that 4 proteins were expected to be extracellularly localized. Blast analysis of these genes against pathogen-host interaction (PHI) database showed that, *g4132* gene is predicted to secret protein homology to an effector protein in *Rhizoctonia solani*, two genes *g1133* and *g1132* are homology to virulence genes in *Ustilago maydis*, while *g4133* gene secrets a protein of uncharacterized function with no hit.

### 3.9. *In planta* Relative Expression Analysis of the Predicted Effector

**Figure 12:**
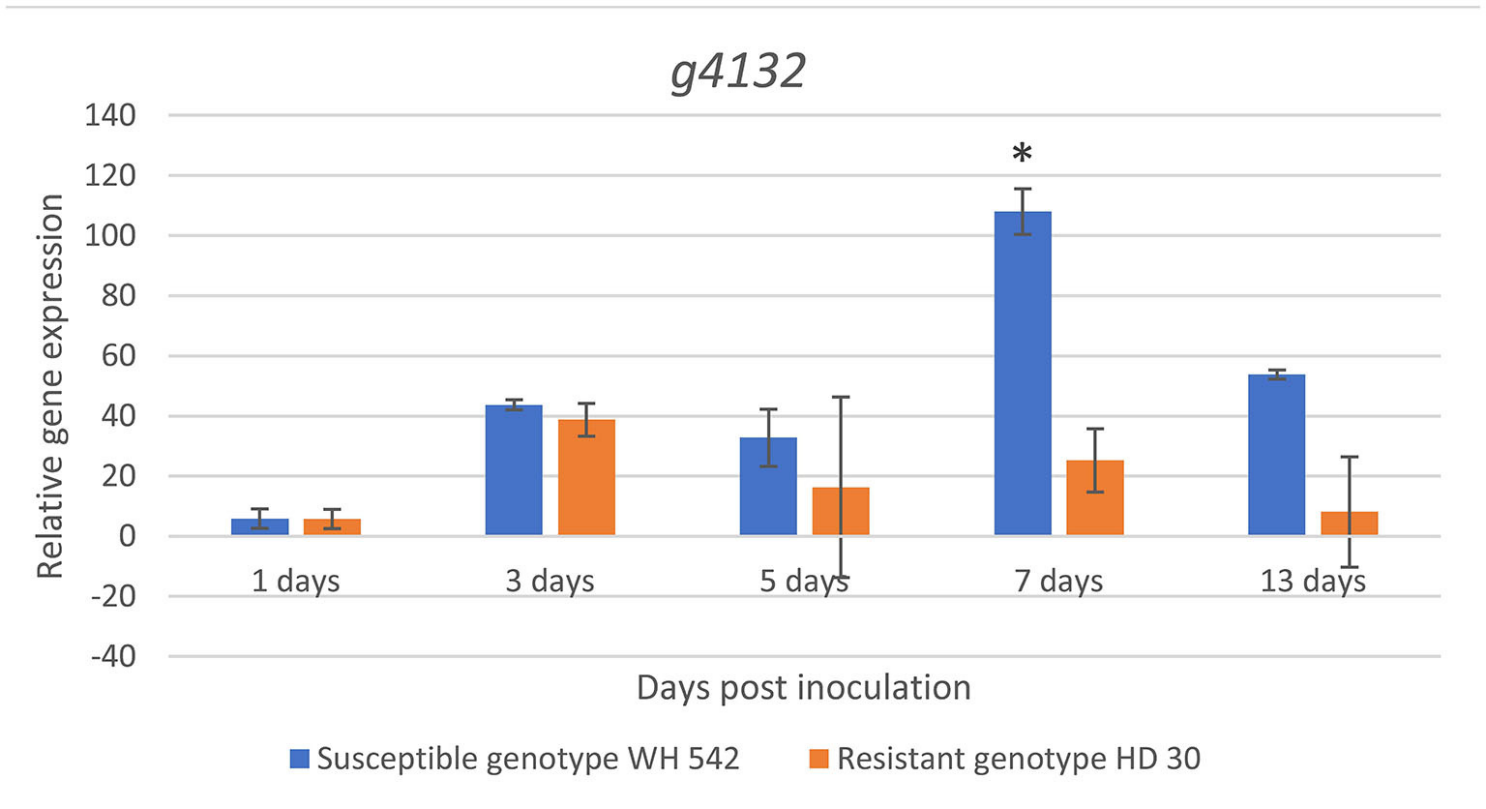
*In planta* relative expression of *T. indica* putative effector gene upon infection in susceptible (WH542) and resistant (HD29) wheat genotypes using qPCR at different time intervals. *Statistically significant expression using unpaired *t*-test.

Expression of the putative effector gene (*g4132*) was found upregulated in both genotypes at 3 days post inoculation (dpi). A sharp statistically significant increase (*p* < 0.05) in the susceptible wheat genotype (WH 542) compared to the resistant genotype (HD30) was detected at 7 dpi reaching its maximum expression 108 fold. After 13 days, the expression decreased up to 53 fold in the susceptible genotype, while the maximum expression in the resistant genotype (HD30) was 38 fold at 3 dpi (Figure 12).

## 4. Discussion

Karnal bunt is a complicated, economically significant, and difficult-to-manage fungal plant disease that affects numerous wheat-growing regions around the world. Effective disease management strategy necessitates knowledge of the pathogen's pathogenicity profiles. Genotyping by sequencing can yield a large number of SNP markers, which can be used to perform powerful analysis, especially when combined with phenotyping data in GWAS (Kim et al., 2016), which allow for the discovery of specific markers linked to virulence genes without the need for population mapping studies (Soto-Cerda and Cloutier, 2012). In the present investigation, genotyping by sequencing 39 *T. indica* isolates using the ddRAD technique was carried out to investigate the genetic variation among isolates, and revealed the presence of 41,473 SNPs exhibiting the high genetic variability among isolates. The population of *T. indica* in India was divided into three subpopulations, each with genetic mixing of several genotypes, revealing the presence of common ancestry between the studied isolates or the result could be influenced by the small size of the population used in this study (Saleh et al., 2014). Previous research revealed that the majority of genetic variation is caused by genetic mutations, parasexual recombination in fungus, and natural or artificial selection pressure (Saleh et al., 2014). One of these variables could be to blame for the observed genetic variety among *T. indica* isolates, where substantial levels of nucleotide polymorphisms were discovered in the 39 isolates tested. The cluster pattern, on the other hand, did not correspond to the pathogenicity of each *T. indica* isolate. Similarly, earlier research has found no link between populations, virulence, and location, which could be due to gene or genotype flow (Rajalakshmi et al., 2017; Korinsak et al., 2019). Our study focuses on gaining a deeper knowledge of *T. indica* pathogenicity. Because *T. indica* is a quarantine fungal pathogen, it is critical to avoid creating new virulent genotypes by artificial sexual reproduction. Thus, rather than using artificial sexual reproduction, we used genome-wide association mapping to find new markers linked to virulence. The trait association mapping study indicated a substantial number of markers (*n* = 13) related to virulence, the location of the retrieved markers on the genome was located, and BLAST analysis validated the homology of the sequences surrounding each SNP (250 Kb upstream and downstream). However, the majority of the markers found were in genes encoding putative proteins whose translation products are still unknown. According to a previous study, the genome of the *T. indica* has more than 10,113 genes, 31.2% of them remaining unannotated (Gurjar et al., 2019). As a result, it is not unexpected that the bulk of the markers found were simply hypothetical protein hits. A total of 12 genes encoding putative virulence factors were identified upon genes annotation. *g1132* and *g1133* genes encode a chitin deacetylase which enables hydrolysis and is involved in the carbohydrate metabolic process. Previously, it was discovered that chitin deacetylase plays a role in virulence *via* direct deacetylation of chitin oligomers, which contributes to lysine motif (LysM)-containing receptor perception in the host, inducing ligand-triggered immunity. This has been observed in a variety of fungal and bacterial pathogens (Gao et al., 2019). *g1135* gene encoding a tRNA 2-thiolation plays a role in pathogenicity by intermediating the induction of some pathogenicity related factors in response to the environmental conditions (San Koh and Sarin, 2018). *g1127* gene encoding a UDP glycosyltransferase (UGT), this enzyme enables the transfer of glucose into glycogen. Recent studies have reported the role of glycogen in pathogen colonization and virulence. The breakdown of glycogen may play an important role in the interactions between the host and pathogens which accumulate glycogen throughout their life cycles (He et al., 2018). *g3612* gene encodes a methyltransferase, which is found to play a role in plant defense reactions suppression, but information on how this is achieved is scarce

(Ludwig-Müller et al., 2015). *g4366* gene encoding an RNB domain-containing protein, it was shown that the adaptability of some pathogens during the infection process is depending on this enzyme's plasticity. It was also reported to be important in eukaryotic cell adhesion and invasion (Matos et al., 2014). *g4527* gene encoding a RING-type domain-containing protein, which manipulates host immune function through promiscuous interactions with numerous proteins evolved during specialized parasite-host interactions (Zhao et al., 2019). *g8485* gene encoding an extracellular carboxylic ester hydrolase, produced by many bacterial pathogens and have been shown to be important for virulence of some pathogens, suggesting that it may play important roles in nutrient utilization and tissue invasion (Xie et al., 2008). *g740* gene encoding an alpha/beta-hydrolase, it was discovered that infection causes it to be induced in *Sclerotinia sclerotiorum*, playing a role in lipid degradation (Seifbarghi et al., 2017). *g4487* gene encoding a cellulase domain-containing protein, which is an important factor in disease development. Several studies support the involvement of cellulases in pathogenesis (Jones and Ospina-Giraldo, 2011). *g4362* gene encoding a thioredoxin domain-containing proteins have the potential to functionally regulate numerous plant immune signaling proteins (Mata-Pérez and Spoel, 2019). *g6053* gene encoding a peptidase M43 domain-containing protein, which is a degrading enzyme used by several pathogens to breakdown host macromolecules, providing nutrition for the pathogen. Moreover, fungal peptidases can inactivate or modify protein components of the host defense machinery and ultimately suppress defense responses (Krishnan et al., 2018). Expression variation of the candidate effector gene (g4132) showed high upregulation in its expression upon infection during host-pathogen interaction in a susceptible host as compared to a resistant host, starting at 3 days post inoculation (dpi), indicating that the pathogen needs few days to establish a relationship with the host (Singh et al., 2020), the expression level continued to increase reaching the expression peak at 7 dpi. The expression pattern of this gene was in accordance with earlier investigations of the pathogen infection (Aggarwal et al., 1994). This analysis will open new prospects in understanding the nature of the pathogen host interaction by using gene expression levels in elaborating the used pathogenic mechanism which will aid in the development of management measures.

This is the first study to use association mapping to identify SNP sites related to virulence genes in *T. indica*. The information gained using this approach will be valuable in future studies, due to narrowing down the genomic regions including virulence genes without having to map the population or undergoing functional analysis. Especially that *T. indica* is very slow growing on artificial media and it is pathogenesis mechanisms is still complex due to the long survival of teliospore in soil and genetic recombination immediately before infection which happens upon mating of two compatible sporidia (Gurjar et al., 021b). These findings will improve the knowledge of *T. indica*-wheat molecular interaction, and enhance the breeding program for durable Karnal bunt resistance. To our knowledge, there have been only two other pathogens apart from this pathogen that have been studied using genotyping by sequencing for population analysis and genetic variability, both of them were performed on ascomycetes fungi *Magnaporthe oryzae* and *Verticillium dahliae* (Rafiei et al., 2018; Korinsak et al., 2019). Genotyping by sequencing was carried out on several isolates of *V. dahliae* and yielded 26,748 single nucleotide polymorphisms (SNPs), widely distributed throughout the genome, which can be used in specific diagnostic markers development, showing the usefulness of using genotyping by sequencing as a high density genotyping tool (Milgroom et al., 2014). In our study, genotyping of 39 *T. indica* isolates yielded 41,473 filtered, high-quality single nucleotide polymorphism, widely dispersed across the genome, offering more in-depth genotyping and association mapping analyses. This is the first time such a large number of SNPs have been reported in this field of study, proving the utility of employing genotyping by sequencing in order to carry out GWAS for the identification of virulence gene markers. This investigation shed light on unique gene functions linked to Karnal bunt virulence that would otherwise go unnoticed by standard experimental analyses. Moreover, it might be possible to identify significantly associated genes with cultivar resistance using this method. Further research is still needed to discover how resistance is conditioned in resistant cultivars and the functions of the identified genes in virulence.

## Data Availability Statement

The data presented in the study are deposited in the NCBI, Sequence Read Archive (SRA) repository, accession number PRJNA796749 (https://www.ncbi.nlm.nih.gov/bioproject/PRJNA796749).

## Author Contributions

MG and MSh contributed to the conception and design of the study. MSh did all the experiments, the result analysis, and wrote the first draft of the manuscript. MSh, MG, RA, RG, NK, MSa, and FK reviewed the manuscript. MG, BB, and FK were in charge of overall direction and planning of research work. SA and TK provided technical assistance. All authors contributed to the manuscript, read, and approved the submitted version.

## Funding

The financial support received from the ICAR-Consortium Research Platform on Genomics (ICAR-G/CRP-Genomics/2015-2720/IARI-12-151) for this research study.

## Conflict of Interest

The authors declare that the research was conducted in the absence of any commercial or financial relationships that could be construed as a potential conflict of interest.

## Publisher's Note

All claims expressed in this article are solely those of the authors and do not necessarily represent those of their affiliated organizations, or those of the publisher, the editors and the reviewers. Any product that may be evaluated in this article, or claim that may be made by its manufacturer, is not guaranteed or endorsed by the publisher.

## Supplementary Material

Supplementary Table S1 Isolates collection location and virulence analysis.

Supplementary Table S2 Sequencing clean data summary. Number of reads, %GC, Mean Read length.

Supplementary Table S3 Genes and Description.
